# Towards the patient-specific design of flow diverters made from helix-like wires: an optimization study

**DOI:** 10.1186/s12938-016-0257-z

**Published:** 2016-12-28

**Authors:** Mingzi Zhang, Hitomi Anzai, Bastien Chopard, Makoto Ohta

**Affiliations:** 1 Graduate School of Engineering, Tohoku University, Katahira 2-1-1, Aoba, Sendai, Miyagi 9808577 Japan; 2 Frontier Research Institute for Interdisciplinary Science, Tohoku University, Katahira 2-1-1, Aoba, Sendai, Miyagi 9808577 Japan; 3 CUI, Department d’Informatique, University of Geneva, 7 route de Drize, 1227 Carouge, Switzerland; 4 Institute of Fluid Science, Tohoku University, Katahira 2-1-1, Aoba, Sendai, Miyagi 9808577 Japan

**Keywords:** Intracranial aneurysm, Flow diverter, Design optimization, Computational fluid dynamics

## Abstract

**Background:**

Flow diverter (FD) intervention is an emerging endovascular technique for treating intracranial aneurysms. High flow-diversion efficiency is desired to accelerate thrombotic occlusion inside the aneurysm; however, the risk of post-stenting stenosis in the parent artery is posed when flow-diversion efficiency is pursued by simply decreasing device porosity. For improving the prognosis of FD intervention, we develop an optimization method for the design of patient-specific FD devices that maintain high levels of porosity.

**Methods:**

An automated structure optimization method for FDs with helix-like wires was developed by applying a combination of lattice Boltzmann fluid simulation and simulated annealing procedure. Employing intra-aneurysmal average velocity as the objective function, the proposed method tailored the wire structure of an FD to a given vascular geometry by rearranging the starting phase of the helix wires.

**Results:**

FD optimization was applied to two idealized (S and C) vascular models and one realistic (R) model. Without altering the original device porosity of 80%, the flow-reduction rates of optimized FDs were improved by 5, 2, and 28% for the S, C, and R models, respectively. Furthermore, the aneurysmal flow patterns after optimization exhibited marked alterations. We confirmed that the disruption of bundle of inflow is of great help in blocking aneurysmal inflow. Axial displacement tests suggested that the optimal FD implanted in the R model possesses good robustness to tolerate uncertain axial positioning errors.

**Conclusions:**

The optimization method developed in this study can be used to identify the FD wire structure with the optimal flow-diversion efficiency. For a given vascular geometry, custom-designed FD structure can maximally reduce the aneurysmal inflow with its porosity maintained at a high level, thereby lowering the risk of post-stenting stenosis. This method facilitates the study of patient-specific designs for FD devices.

## Background

Flow diverter (FD) intervention has become increasingly attractive for the treatment of wide-neck and fusiform intracranial aneurysms (IAs), which has been studied intensively by many groups [1–4] in recent years. Meanwhile, clinical follow-ups revealed that FD recipients might incur post-stenting complications such as delayed aneurysm ruptures and post-stenting stenosis [5–7].

The conventional, commercially available FD devices such as Pipeline embolization device (PED; Irvine, CA, USA) and SILK (Balt, Montmorency, France) are constructed by homogeneous helix-like wires. The porosity of FD device is associated with post-stenting stenosis as suggested by prior studies [8], since a high metal-to-arterial tissue ratio resulted from low device porosity may pose the risk of vascular injury. Animal experiments, on the other hand, have confirmed that a lower device porosity promotes a complete thrombotic occlusion of an aneurysm [9, 10]. Therefore, simply modifying FD wires by decreasing or increasing the porosity may result in an increased risk of post-stenting stenosis or long-term thrombosis formation. To accelerate thrombotic occlusion and avoid post-stenting stenosis, a possible solution may be the application of patient-specifically tailored FDs with porosity maintained at a high level. Attempts of introducing optimization to FD structures have been made to improve the flow-diversion efficiency [11–13]. However, a practical optimization strategy that can be feasibly applied to the conventional FDs has not yet been developed.

In this study, we demonstrate an automated optimization method on a conventional, homogeneous, helix-like FD to adapt its wire structure to an assigned aneurysm. The proposed optimization was designed to rearrange the starting phases of FD wires, so that the original device porosity was kept to maintain the metal-to-arterial tissue ratio. After the FD structure with the highest flow-diversion efficiency was identified, its robust performance was then investigated by axial displacement test.

## Methods

**Fig. 1:**
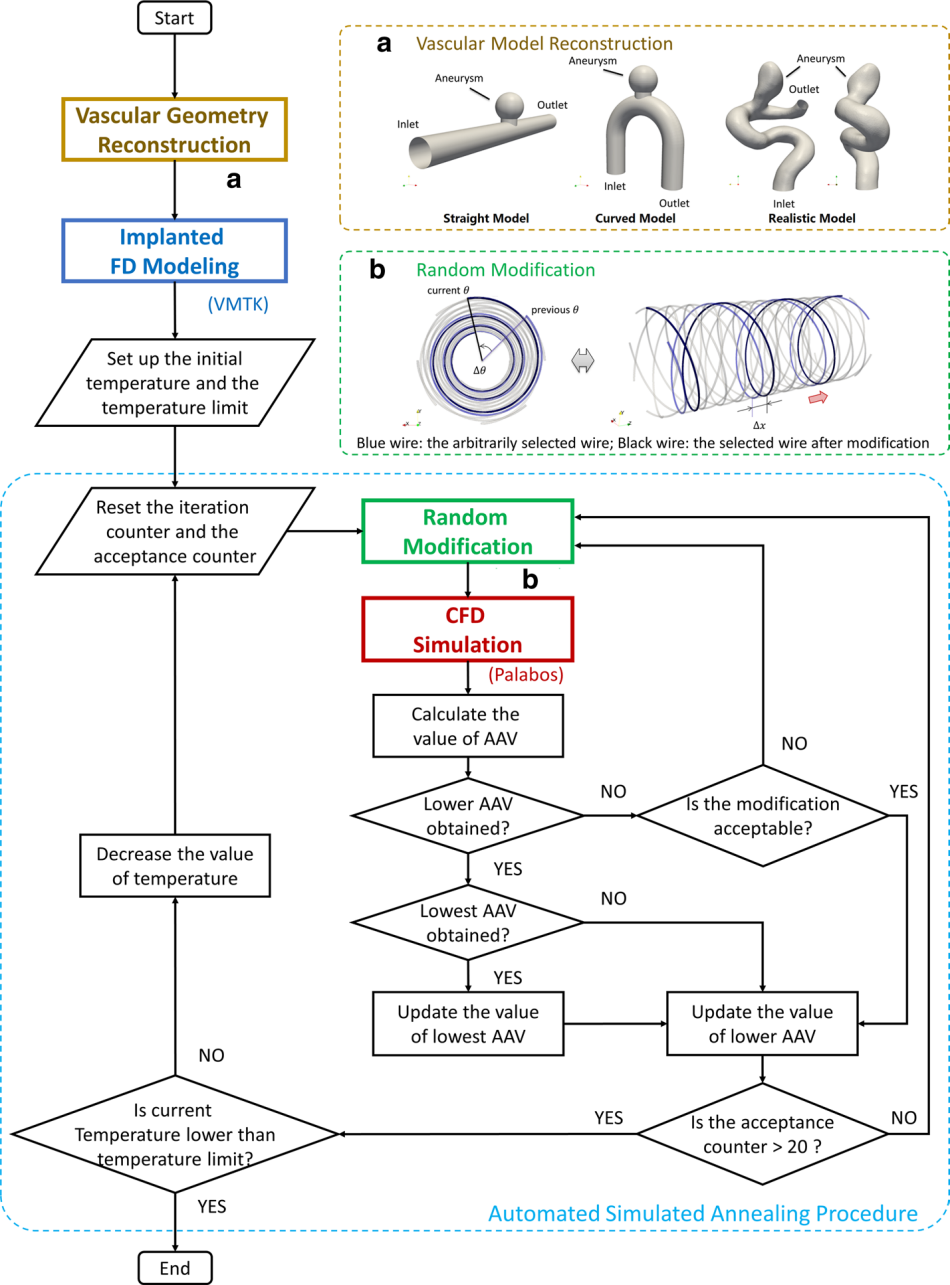
The schematic of the proposed optimization procedure. *AAV* aneurysmal average velocity

Figure 1 shows the scheme of our proposed optimization method, which includes vascular model reconstruction, FD modeling, random modification, computational fluid dynamic (CFD) simulation, and a simulated annealing (SA) procedure.

### Vascular model

Aneurysmal local hemodynamics is sensitive to the morphological characteristics of the parent artery. Thus, three vascular geometries were used to investigate the proposed optimization method under various hemodynamic conditions.

### Idealized aneurysm geometry

Two idealized aneurysm models—the Straight (S, Fig. 1a) model and the Curved (C, Fig. 1a) model—were constructed; for both models, the aneurysmal diameter (*D*) was 4.8 mm, the neck diameter (*N*) was 2.8 mm, and the arterial diameter (*d*_1_) was 3.5 mm. The curvature radius (*r*) of the C model was 6.0 mm [14].

### Realistic aneurysm geometry

The 3D patient-specific geometry of a human internal carotid artery (ICA) with an aneurysm was reconstructed (R model, Fig. 1a). The parent artery had an inlet diameter (*d*_2_) of 3.8 mm [15].

### FD model

Commercially available, braided FD devices are usually made of helix-like woven wires with uniform structural arrangements. In this study, the FD was assumed to comprise eight helices (four clockwise and four counterclockwise); wire thickness and width were both 50 μm. Each helix trajectory of the deployed FD was individually described by the following equations:

1 $$ {\text{Clockwise}}: \left\{ {\begin{array}{*{20}l} { x = \left[ {R + r*sin\left( {\omega_{\alpha } + \theta_{n} } \right)} \right]*\cos \left( {\omega_{\beta } } \right)} \\ { y = \left[ {R + r*sin\left( {\omega_{\alpha } + \theta_{n} } \right)} \right]*sin\left( {\omega_{\beta } } \right)} \\ {z = r*\cos \left( {\omega_{\alpha } + \theta_{n} } \right)} \\ \end{array} } \right. $$

2 $$ {\text{Counterclockwise}} : \left\{ {\begin{array}{*{20}l} { x = \left[ {R + r*cos\left( {\omega_{\alpha } + \theta_{m} } \right)} \right]*\cos \left( {\omega_{\beta } } \right)} \\ { y = \left[ {R + r*cos\left( {\omega_{\alpha } + \theta_{m} } \right)} \right]*sin\left( {\omega_{\beta } } \right)} \\ {z = r*\sin \left( {\omega_{\alpha } + \theta_{m} } \right)} \\ \end{array} } \right. $$

where *r* and *R* denote the radius and curvature radius of a helix, respectively; $\omega_{\alpha }$  and $\omega_{\beta }$ are parameters associated with the length and pitch of a helix, respectively; and $\theta_{n }$ or $\theta_{m }$ indicates the starting phase of a helix. To imitate FD devices with uniform structures, the starting phase conditions satisfied

3 $$ \theta_{n} = 2\left( {n - 1} \right) \cdot \frac{\pi }{4}   \,{\text{and }} $$

4 $$ \theta_{m} = 2\left( {m - \frac{1}{2}} \right) \cdot \frac{\pi }{4}, $$

where $n\,or\,m\, \in \,(1, \,2,\, 3,\, 4)$ indicates the sequence of either the four clockwise (*n*) or the four counterclockwise (*m*) helical subsets.

The helix radius *r* varied with respect to the discrete points along the centerline of the parent artery and was associated with the maximum inscribed sphere radius (MISR) corresponding to each point. The coordinates of discretized points and their corresponding MISRs were measured using the open library vascular modeling toolkit VMTK v1.2 [16]. Given the above FD parameters, the FD porosity can be calculated according to a previously defined equation [17]

5 $$ {\text{Porosity}}\,\left( \% \right)\, = \,\frac{{S_{total} - S_{metal} }}{{S_{total} }}\, \times \,100 $$

where *S*_*total*_ and *S*_*metal*_ denote the surface area of the FD’s generalized cylinder and the FD’s metal wires, respectively. The device porosity was fixed at 80% in this study.

### Random modification

Random modification was designed to modify an FD structure while maintaining its original device porosity. During each stage of random modification, one of the eight helices was arbitrarily chosen, and a stochastic variable $\Delta \theta \in \,\left( { - \frac{\pi }{8},\frac{\pi }{8}} \right)$ was then added to the starting phase *θ* (either *θ*_*n*_ or *θ*_*m*_) of the selected helix:

6 $$ \theta_{current} \, = \,\theta_{previous} \, + \,\Delta \theta $$

In this manner, the modification resulted in the axial displacement of the arbitrarily selected helix along the centerline of the parent artery, whereas the device porosity remained unchanged (Fig. 1).

### Hemodynamic simulation

The open source library Palabos (version 1.4) [18] based on lattice Boltzmann method (LBM) was used as the CFD solver for its high flexibility and parallelism. LBM is a mesoscopic approach which exhibits good agreement along with its similar numerical stability to other CFD tools [19–21]. In LBM, fluid is described in terms of the density distribution *f*_*i*_ (*r*, *t*) of idealized fluid particles moving and colliding on a regular lattice. These collision-propagation dynamics can be written as

7 $$ f_{i} \,\left( {r + \Delta tv_{i} ,t + \Delta t} \right)\, = \,f_{i} \,\left( {r,t} \right) + \frac{1}{\tau }\,\left( {f_{i}^{eq} - f_{i} } \right), $$

where *f* ^*eq*^ and *τ* are the local equilibrium distribution and relaxation time, respectively [22]. Because LBM uses a Cartesian mesh, manual computational grid generation was avoided, allowing the optimization procedure to be entirely automated.

A previous study suggested that the peak value of hemodynamic parameters computed for pulsatile flow matches those of the corresponding steady flow [23]. Therefore, we performed steady flow simulation with standard D3Q19 lattice topology [22] in consideration of hundreds of CFD simulation steps in the following optimization procedure.

The bounce-back rule was used to impose the no-slip boundary conditions as well as to define FD wire structures. The spatial discretization $(\Delta r)$ was set at 0.05 mm after sensitivity tests, in which we found no obvious differences in the intra-aneurysmal average velocity and flow patterns after doubling the lattice grid resolution. The numbers of fluid cells for the S, C, and R models were 3.57 × 10^6^, 3.01 × 10^6^, and 4.06 × 10^6^, respectively.

The blood flow was assumed to be an incompressible Newtonian fluid. To reach the same Reynolds number (*Re*) of 200, velocity was defined as parabolic profiles at inlets of 0.23, 0.23, and 0.21 m/s for the S, C, and R models, respectively. A constant pressure boundary was imposed at the outlets. The density and kinematic viscosity were assumed to be constant at 1040 kg/m^3^ and 4.0 × 10^−6^ m^2^/s, respectively. The kinetic viscosity of the lattice (*ν*_*LB*_) was chosen as 0.012, giving a relaxation time *τ* of $(6\nu_{LB} + 1)/2\, = \,0.536$. We assumed that the simulation had reached a convergent state when the change in the average energy of fluid cells was less than 10^−6^ kg m^2^/s^2^.

### Simulated annealing

To control the random modifications progressing towards the optimal solution, a SA procedure (Fig. 1) was implemented to identify the FD structure with the lowest intra-aneurysmal average velocity within a certain range of temperature drop [24, 25]. We selected intra-aneurysmal average velocity as the objective function of SA because of its possible correlation to thrombotic occlusion [26].

Optimization began with the homogeneous FD structure and was completed when the lower temperature limit was reached. The initial and lower temperature limits were decided in this manner: (1) Prior to the SA procedure, hundreds of random modifications were performed to calculate the mean alteration of objective function for assigning the initial temperature an acceptance probability of 0.5; (2) the optimization was assumed to involve 60 decreases in temperature and the lower limits for all cases were calculated accordingly as shown in Table 1. During optimization, the FD structure was first modified, and CFD was then performed to obtain the corresponding intra-aneurysmal average velocity in each stage.

**Table 1:** Initial temperatures and lower temperature limits for the S, C, and R models

	S model	C model	R model
Initial temperature	6.02 × 10^−5^	1.27 × 10^−4^	1.01 × 10^−3^
Lower temperature limit	1.08 × 10^−7^	2.28 × 10^−7^	1.81 × 10^−6^

We used the scalar parallel computing system (SGI UV2000) at the Institute of Fluid Science, Tohoku University. The computational times for one stage of CFD simulation (using 256 cores) were approximately 30, 45, and 70 min for the S, C, and R models, respectively.

### Axial displacement test (ADT)

The manual operation of FD delivery and deployment leads to an uncertain axial displacement during the interventional procedure. An axial displacement test (ADT) is designed to investigate the robustness of a given FD structure. For a given FD structure, we sequentially added the variable $\theta_{\Delta }$, ranging from −π to π, to the starting phase *θ* of each helix to mimic the axial displacement along the centerline of the parent artery. A CFD simulation was subsequently performed to calculate the difference in velocity resulting from the displacement.

### Flow reduction (FR) rate

To quantitatively evaluate the flow-diversion efficiency of a given FD structure, a flow reduction (FR) rate index was introduced as

8 $$ R_{f} \,(\% )\, = \,\frac{{V_{w/o} - V_{withFD} }}{{V_{w/o} }}\, \times \,100, $$

where *V*_*w*/*o*_ and *V*_*withFD*_ are the intra-aneurysmal average velocities without FD intervention and after FD implantation of a given wire configuration, respectively.

## Results

**Fig. 2:**
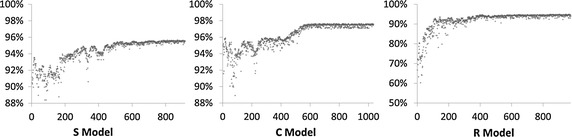
The SA procedure of S, C, and R models, respectively (Vertical axis: *R*_*f*_, horizontal axis: SA iteration)

For each case, optimization was performed until the lower temperature limit was reached; this required 916, 1035, and 976 iterations for the S, C, and R models, respectively. In all three cases, the FR improved markedly during the initial few hundred iterations and then stabilized (Fig. 2). The intra-aneurysmal average velocity without FD intervention and the *R*_*f*_ values after FD implantation with the initial and optimal configurations are shown in Table 2.

**Table 2:** Intra-aneurysmal average velocity and the *R*_*f*_ under non-stent, initial stent, and optimal stent placements of S, C, and R models, respectively

	Non-stent	Initial stent placement		Optimal stent placement	
	*V*_*w*/*o*_ (mm/s)	*V*_*initial*_ (mm/s)	*R*_*f*_ (%)	*V*_*optimal*_ (mm/s)	*R*_*f*_ (%)
S model	2.827	0.269	90.48	0.123	95.65
C model	10.629	0.528	95.04	0.249	97.65
R model	39.309	13.071	66.75	2.025	94.85

**Fig. 3:**
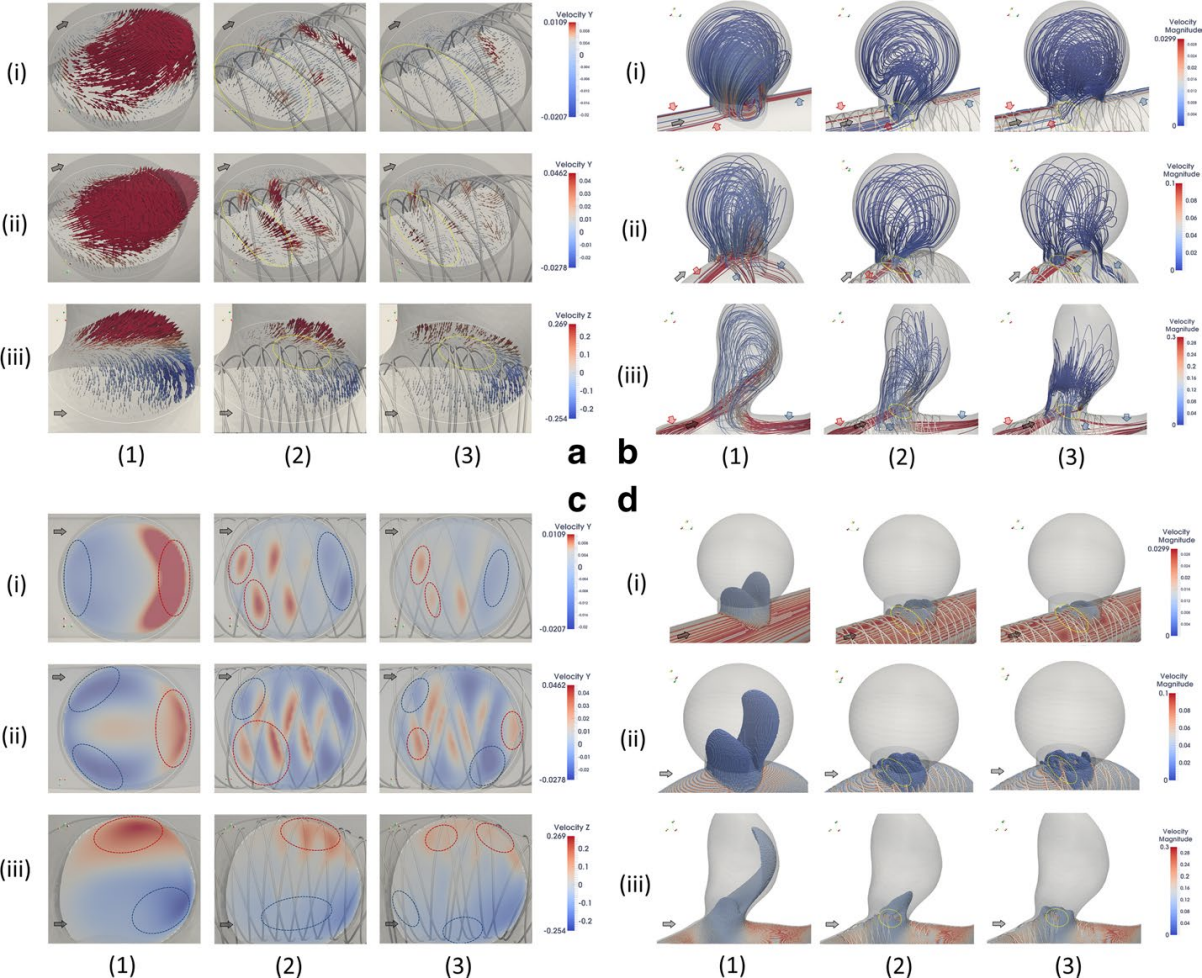
Visualizations of velocity vectors (Block **a**), streamlines (Block **b**), velocity components perpendicular to neck orifices (Block **c**), and ISO-velocity surfaces (Block **d**) of non-stented cases (*Column 1* of each block), initial FD placements (*Column 2* of each block), and optimal FD placements (*Column 3* of each block), respectively; S model, C model and R model are in row (*i*), row (*ii*), and row (*iii*) of each block, respectively

**Fig. 4:**
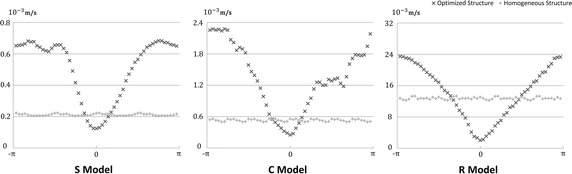
The ADTs of S, C, and R models, respectively (Vertical axis: aneurysmal average velocities, horizontal axis: phase displacements)

Figure 3b, d depict the streamlines (color-coded by velocity magnitude) and iso velocity surface (corresponding to 0.01, 0.015, and 0.1 m/s for the S, C, and R models, respectively) of the three geometries with no stent and stents before and after optimization. Figure 3a, c illustrate the velocity components perpendicular to and velocity vectors generated from the aneurysm orifice. Figure 4 illustrates the intra-aneurysmal average velocity differences with respect to the axial displacements for both the initial and optimized FD structures.

Aneurysmal inflow is observed as a flow bundle entering from the aneurysm orifice. The concept of bundle of inflow (BOI) area describes the inflow feature of an aneurysm [13]. Likewise, the bundle of outflow (BOO) area indicates the region(s) where the bloodstream exits an aneurysm. To demonstrate the unique features of different flow patterns, the red and blue arrows/circles are used in Fig. 3b, c to indicate the BOI and BOO areas, respectively; the yellow circles with dotted lines in Fig. 3a, b, d depict the areas where BOI concentrates. The black arrows in all figures are used to identify the flow directions.

### S model

Before FD stent implantation, the flow enters the aneurysm from the distal end and exits through the proximal end. After FD implantation with the initial structure, the symmetric flow distribution was disrupted; the flow circulation inside of the aneurysm sac became irregular and sparse. The BOI area shifted from the central distal end to the proximal end with a negative Y offset; meanwhile, the outflow zone switched to the distal end (Fig. 3c). A marked reduction in both the breadth and magnitude of the BOI was observed for the optimized FD structure (Fig. 3b). Rotational flow circulations were found inside the aneurysm. The optimized wire structure showed a concentration of FD wires inside the BOI area, resulting in a denser strut distribution in the proximal orifice end (Fig. 3a).

### C model

Under the non-stent condition, a strong inflow jet and two outflow jets were observed (Fig. 3d). The BOI was located in the proximal area of the neck, whereas the BOOs were symmetrically dispersed along both sides of the aneurysmal neck. After the initial FD implantation, the magnitude of the velocity of the BOI decreased considerably (Fig. 3b), and the outflow bundles were disrupted. For the optimized FD, the velocity magnitude of the BOI was further reduced. A denser strut distribution inside the BOI region was observed in the optimized case (Fig. 3a).

### R model

Compared with the S and C models, the non-stent R model has a strong and sharp inflow jet, as seen in Fig. 3d. The bloodstream flows into the deep aneurysm sac through the orifice and circulates inside the cavity, finally flowing out of the aneurysm as a wide and strong BOO. After the FD implantation of the initial structure, the width and velocity magnitude of BOI were reduced; both the size and volume of the isovelocity surface decreased (Fig. 3d). After the implantation of the optimized FD, the flow circulation was drastically modified. The depth traveled by the inflow jet into the aneurysm sac decreased, and rotational flow circulation was clearly observed inside the sac (Fig. 3b). The sac circulation and parent flow were further separated, and both BOI and BOO were split into two weak and thin streams (Fig. 3c). Similarly, the wire concentration inside the BOI area of the optimized FD structure can be seen in Fig. 3a.

## Discussion

In this study, we have demonstrated an optimization approach to improve the flow-diversion efficiency of conventional homogeneous FD stents. Our optimization involves rearranging the starting phases of homogeneous helix wires, thereby modifying FD structure without altering its porosity, which finally enables the optimal wire configuration to maximally block the aneurysmal inflow.

### Selecting the modification parameter for optimization

In consideration of the possible correlation between porosity and post-stenting stenosis, we chose the starting phase *θ* of each FD helix as the modification parameter.

Previous studies on FD structure optimization have introduced various modification parameters to improve flow reduction effectiveness. Anzai et al. [13] modified the configurations of the strut segments within the aneurysmal neck domain and found that the flow reduction effectiveness was improved when the stent porosity was strictly maintained at 80%, while the isolated and disconnected strut segments denied the manufactural possibility of the optimal FD structure. Lee et al. [12] used strut size and gap spacing as modification parameters and obtained relatively optimal designs. However, the porosity of FD devices cannot be precisely controlled during modification.

Theta is one of the parameters required in FD design and manufacturing process. For design optimization, using *θ* as modification parameter could maintain the device porosity at a pre-defined value. Our results demonstrate (Fig. 2) that modifying *θ* effectively improves the flow reduction rate of a conventional homogeneous FD structure.

### Objective function of optimization and SA procedure

We chose intra-aneurysmal average velocity as the objective function. Anzai et al. [13, 27] used average velocity, while Srinivas et al. [11, 12] used average velocity and vorticity, and Janiga et al. [28] used wall shear stress for optimization.

It should be noted that the selection of objective function remains a controversial issue. Corbett et al. [29] reported an in vitro study that thrombosis could occur in a specific threshold of shear stress or shear rate using bovine blood. A review by Moiseyev et al. [30] revealed that the shear-induced activation of platelets is a basic element for blood coagulation. On the other hand, Janiga et al. [28] described that the flow reduction within an aneurysm is relevant to wall shear stress, and recently, Chung et al. [26] showed that the average velocity in the aneurysm may be related to a shorter period of aneurysm occlusion. Revealed by these results, the average velocity seems to be correlated with thrombotic occlusion. However, further in vivo study is still indispensable for confirming its relevance in clinical practice.

We employed SA procedure to identify a global optimum for the average velocity as shown in Fig. 2. To prevent optimization from resulting in a local optimum, modifications to FD structures with inferior flow diversion performance might also be accepted according to our pre-defined cooling schedule. Our optimization approach could accept other objective functions, as long as the initial temperature and cooling schedule are well established [24, 25].

### FD design with inhomogeneous wire structure

Our optimization generated modified FD designs with inhomogeneous wire structure. Similarly, previous studies also reported the benefits of inhomogeneous and asymmetric FD designs. Rudin et al. [31] showed that a local low-porosity design can decrease flow velocity inside an in vitro model. By animal experiments, Ionita et al. [32] showed the good performance of asymmetric stents in occluding rabbit elastase aneurysms. These studies suggest the possibility of using inhomogeneous device to achieve favorite treatment outcomes. In this study, we demonstrated how a conventional homogeneous FD can be tailored to an inhomogeneous one by merely changing the values of ‘*θ*’, which is applicable to be modified without affecting the manufacturing possibility.

In addition, our optimization results have revealed a practical approach for the conventional homogeneous FD devices to improve its flow diversion efficiency, that is, compacting FD wires into BOI areas during deployment may achieve a marked difference in blocking the aneurysmal inflow.

### Robust performance of the optimal devices

We performed ADTs to investigate the robustness of the wire structures. The flow reductions achieved by the optimized FDs were greater than those obtained using the homogeneous FDs within displacement ranges of −0.25 to 0.25, −0.5 to 0.25, and −0.75 to 0.75 mm for the S, C, and R models, respectively (Fig. 4). It is indicated that a homogeneous wire configuration can nonspecifically prevent a strong inflow jet from passing through an aneurysm orifice; however, its flow-diversion efficiency is inferior to that of the optimal wire configuration when the device was desirably deployed.

The robustness of an optimized FD is associated with the wire coverage of an aneurysm orifice. After homogeneous FD implantation, if the BOI areas are axially distributed along the orifice (e.g. R model, Fig. 3c), the robustness of an optimized FD might be superior. In contrast, if the BOI areas are radially distributed (e.g. S and C models, Fig. 3c), the optimized FD robustness could be inferior. The optimized FD wires concentrate in BOI regions, whereas only a small number of wires are assigned in the remaining areas where large holes can be found. When axial displacement occurs, the inflow jet may cross the orifice through the holes, causing considerable fluctuations of *R*_*f*_.

The robustness of an optimized FD may also relate to the shape of an BOI area. When BOI is strong and concentrates in a small region (e.g. C model, Fig. 3c), axial displacements may result in jet flow entering aneurysm cavity through areas with less wires. It is implied that the BOI characteristics of the FD recipient needs to be investigated before an optimized device can be applied.

## Limitations

This study has several limitations. We applied steady flow and Newtonian fluid assumptions to reduce the computational cost, since the objective of this study is to develop a feasible and manufacture-oriented optimization approach for FD device. When computational cost is no longer a problem, the setting is readily changed by adopting time-dependent boundary conditions and a non-Newtonian rheology in the LB solver.

Another limitation from the viewpoint of clinical practice is that sending the optimized FD to the aneurysm location could be a challenge for interventionists, since a larger device displacement after deployment may considerably increase the aneurysmal inflow. This might be solved in the future by embedding a reliability test into the optimization loop to achieve improved stability.

It should be noted that we addressed merely the hemodynamic factors that may affect the FD performance, while the mechanical and material properties of the modified FDs have not been investigated. In future work, we plan to include these parameters as a part of objective functions for optimization to improve FD’s hemodynamic compatibility.

## Conclusions

A practical optimization method for commercially available helix-wire FDs was developed in this study. By rearranging the starting phase of each helix subset, the structure of FD can be tailored to efficiently block the inflow for a patient-specific aneurysm. Using this optimization method, three optimized FD structures with unchanged device porosity were obtained corresponding to three different vascular geometries. The developed method potentially enhances the study of the patient-specific design of FD devices.
